# The Cæsarian Section*Read before the Buffalo Obstetrical Society.

**Published:** 1884-12

**Authors:** Stephen Y. Howell


BUFFALO

Medical and Surgical Journal.

VOL. XXIV. DECEMBER, 1884. No. 5.

### Original Communications.

### THE CÆSARIAN SECTION.*

BY STEPHEN Y. HOWELL, M. A., M. D.

Richard Volkmann, one of Germany's most brilliant surgeons, in his address on "Modern Surgery,"† delivered in London on the 8th of August, 1881, before the International Medical Congress, pronounced a most eloquent eulogium upon the author of modern antisepsis. "Never," said he, "has a surgical discovery been made which conferred upon suffering humanity even a tithe of the blessings which have followed in the wake of this one * * * ; and England may well be proud that 'tis one of her sons whose name is so inseparably linked to this, the greatest stride which surgery has ever taken."

And, certainly, in the few years which have since elapsed, there has transpired nothing which detracts in the slightest degree from the truth of his remarks; on the contrary, the beneficent influence of Joseph Lister's work is extending in ever widening circles, invading every department of medicine where the aid of the knife is invoked, and whispering words of comfort and hope to thousands of despairing mortals in every civilized land.

* Read before the Buffalo Obstetrical Society. † Samml. Klin. Vorträge, No. 221.

Nor has our own science been backward in availing herself of the benefits of Listerism. Operative obstetrics has steadily advanced, *pari passu*, with other branches of surgery; and operations, whose very names were well-nigh synonymous with death only two decades since, are now performed with comparative immunity from risk, and rank as feasible means of escape for both mother and child, when nature or disease has obstructed the parturient passages of the gravid woman to such an extent that spontaneous delivery is out of the question, and the forceps or version are of no avail. In view, then, of its enhanced usefulness and probable popularity in the near future, I deem no apology necessary before directing your attention, for a few moments, to the Cæsarian operation and some of its modifications.

Lost, as it were, in the night of time, the origin of this operation has not, as yet, been precisely ascertained. In the mythological ages 'twas said that a fœtus, the son of Jupiter, was extracted from the belly of Semele by Mercury; and the Romans made the same statement concerning Æsculapius, the god of medicine, who was rescued by his father, Apollo, after the mother had already been placed on the funeral pile, which was destined to consume her. Virgil, also, says that Lycus came into the world in the same manner; and, according to Pliny, Scipio Africanus and Manlius were thus spared to the Roman State.

Indeed, the Greeks and Romans seem to have attached great stress to the performance of the operation upon women who died in advanced stages of pregnancy, or during labor.

Thus Numa Pompilius decreed, in the *lex regia*,* that the fœtus should always be removed by abdominal section before the burial of the mother; and the Italian laws, too, made this necessary; while, as late as the middle of the eighteenth century, the King of Sicily sentenced to death any physician who was guilty of the sin of omission in this respect. M. Mansfield, in

* Digest., lib. IX., tit. VIII., l. 2, et lib. I., tit. V., etc.

a work from which an excerpt appeared in the *Bulletin des Sciences Médicales*, attempts to prove that the Cæsarian operation was practiced even by the Jews; and it is stated in the Talmud and the Mischajoth, that a child born by the section of the belly has not the right of primogeniture.

As to the etymology of the term, Cæsarian section, Guy de Chauliac, who, apparently, was the first to describe the operation, believed it to be derived from Julius Cæsar, founding his opinion upon the familiar passage of Pliny: *Auspicatius, enecta parente, gignuntur, sicut Scipio Africanus prior natus, primusque Cæsus, cæso matris utero, dictus; qua de causa, Cæsones appellati*, etc., to which we've already referred; while others declare that the children thus saved were called Cæsar, and that the name was later adopted as a family patronymic.

Thus history, both ancient and modern, affords abundant encouragement for the performance of post mortem gastro-hysterotomy, admonishing us at the same time, however, to operate with care, when it is not absolutely certain that death has supervened. Thus Van Swieten and Baudelocque mention three cases of apparent death, in which recovery from a lethargic state ensued just before the operation was commenced, and Peu relates an instance which is still better calculated to excite caution.

He had begun his incision, when the woman gave a shudder, accompanied with grinding of the teeth and a movement of the lips; but, whether these phenomena sufficed to secure a postponement of the proceedings, history saith not. Sudden death, then, in late pregnancy or during labor, would call for the prompt release of the fœtus, either *per vias naturales*, were this expedient, or by the abdominal section.

But, in resorting to the latter alternative, we should not be guided by the quaint advice of Guy de Chauliac, who says: "Let the woman be opened with a razor along the left side, inasmuch as that part is freer, on account of the liver," but, rather, proceed as when operating on the living patient, prepared, as far as may be, for the worst.

Duer's tables of 55 cases of post mortem Cæsarian section show that forty living children were extracted: 21 in from 1 to 5 minutes after death of mother; 0 between 5 and 10 minutes; 13 in from 10 to 15 minutes; 2 in from 15 to 23 minutes; 2 after one hour, and 2 after two hours,—results which not only warrant, but which demand legal interference in our own day.

For, could opposition and consequent delay be avoided in such trying junctures by an appeal to the law, not only would the operation be more frequently performed when indicated, but a still greater percentage of children would be spared to lead lives of probable usefulness.

Rousset was the first surgeon who had the temerity to propose the performance of gastro-hysterotomy upon the living woman; and he mentioned seven instances in which it had been resorted to successfully.

Bauhin, too, cited several cases, including that of J. Nufer, a spayer of cattle, who, in 1500, operated successfully upon his own wife, after she had been given over by several midwives. But none of these cases bear a rigid investigation; indeed, the majority of the so-called Cæsarian sections of that time seem to have been operations for the relief of extra-uterine pregnancy.

The first really authentic laparo-hysterotomy was probably performed by Trautmann, of Wittenberg, in the year 1610, upon a case of hernia of the gravid uterus, which is vouched for by Tandler, Sennert and Doering. Considerable time elapsed, however, ere the labors of distinguished men like Levret, and Stein, the elder, served to establish the indications for the new and heroic operation.

But their efforts finally prevailed; and, in spite of the opposition of such men as Paré, Guillemeau and Sacombe; in spite of the wonderful popularity of its great rival, Symphyseotomy,—proposed by the student Sigault, and the occasion of those violent polemics between the Symphysians and Cæsarians from 1777 to the commencement of the present century,—the Cæsarian operation not only flourished, but was scandalously abused as late as 1827. In this year, however, Baudelocque, the younger, scored a point for the obstetricians by inventing the cephalotribe, an instrument which, in its improved form, has been the most active agent in limiting the employment of the Cæsarian section; though its use means the certain death o the child, and the exposure of the mother to a risk, fully equal to that offered by its more dreaded rival, whenever the conjugate diameter measures less than two and three-fifths inches.

But, without stopping now to discuss the various alternatives which present themselves, and simply premising that the Cæsarian section is absolutely indicated when the child is at term and the internal sacro-pubic diameter measures less than two inches, we pass on to a description of the conditions which should obtain and the various steps of the manual operation.

The most fitting time for operating is during the first stage of labor, before the rupture of the membranes, when the os uteri is somewhat dilated and the pains vigorous. If we begin sooner, the uterine contractions, subsequent to the operation and to which so much importance attaches, are defective; while, on the other hand, the previous rupture of the sac enhances the difficulty of the procedure, and renders the prognosis for the child less favorable. For the mother, the exhaustive effects of delay, coupled, possibly, with decomposition of the secretions, render the outlook more gloomy.

In doing a gastro-hysterotomy one proceeds, on the whole, as in simple gastrotomy, taking the most stringent precautions to combine great personal cleanliness with as complete a disinfection of room, utensils, etc., as possible; though the use of the spray during the operation may be omitted. As antiseptic agents are employed carbolic acid, corrosive sublimate and thymol.

The bowels and bladder having been thoroughly evacuated, the patient is anæsthetized, while the parts are being shaved and washed with the sublimate solution (1-1000). Any intestinal folds between the uterus and anterior abdominal wall are stroked to one side, and the organ is firmly held in the median line by an assistant, who places a hand on each side, and renders the abdominal parietes tense over the uterine convexity.

An incision some six inches in length is now made in the linea alba—Mauriceau's method—and carefully carried through the parietal peritoneum in the usual manner; when the uterus appears as a large, red, tense globe from which the omentum, if present, is displaced upwards. So far the operation has slowly and gradually progressed.

But, as soon as the operator begins to sever the fibres of the uterine globe, which distends the parietal aperture, he must proceed swiftly as well as coolly; for, as a rule, the hemorrhage from the walls of that organ is severe,—at times even appalling. Now presence of mind is at a premium! Only one thing can stay the rush of blood, and that is the evacuation of the uterine contents. The fiercer the hemorrhage, then, the quicker must be the cutting, and it is best to rapidly deepen the upper end of the incision till the ovum is exposed, when the fingers are inserted as guides, and the opening is completed with a blunt-pointed bistoury. Should the placenta be implanted in the line of incision, it is peeled from its insertion on one side, rather than severed. Winckel recommends that the fingers of an assistant be hooked into the extremities of the uterine wound, in order that its edges may the more effectually be apposed to the abdominal incision, thus preventing the escape of fluids into the abdominal cavity and the protrusion of viscera.

Now let the operator rupture the membranes, seize the child by the presenting part; or, preferably, by the head, to prevent delay by the rapidly contracting uterus, and quickly extricate it. The placenta is generally loosened by the contraction of the uterus, and is easily removed; though, should it still adhere, it may be carefully separated with the fingers, while the diminishing organ is followed by the hands of the assistant to prevent the prolapse of omentum or intestine.

Child and placenta being removed, the uterus usually contracts promptly, and thus the hemorrhage from the placental site, and the line of incision, now reduced in length from about six to two inches, is well-nigh completely stilled. But the incised uterine surfaces do not come into perfect apposition; the wound gapes, especially the peripheral layers of muscular fibres; and, in order to remedy this and prevent the subsequent escape of fluids into the peritoneal cavity, deep and superficial sutures of silk, catgut (Veit*) or metal are passed, after first cleansing the uterine cavity with a strong solution of carbolic acid, 5 per cent. at least, and inserting a drainage tube, which extends through the cervical canal into the vagina. Another moment calls for the insertion of uterine sutures, and that is the variable state of the organ—now larger and thinner, now smaller and thicker—for which reason it is also well that the sutures should be secured by three or four single knots to prevent their becoming untied. As will readily be seen, however, this alternate contraction and relaxation of the uterine walls tends to loosen the sutures, and, probably, suffers the escape of the lochia, pus and blood through the incision into the peritoneal cavity, where they serve to light up what may prove a fatal peritonitis. And yet, recent successes have demonstrated the fact, that the chances of recovery after Cæsarian section are enhanced by thus closing the uterine wound. In the hope of overcoming this difficulty, however, Drs. Grandesso-Sylvestri and Valentinoldi have employed elastic sutures of rubber, covered with silk, and these seemed to have fulfilled the indications very well. Spencer Wells suggests a continuous suture, which may afterward be withdrawn.†

E. Martin,‡ of Berlin, and Prof. Olshausen,§ of Halle, have favored stitching the uterus to the lower end of the abdominal

---

* Berl. B. z. Geb. u. Gyn., III., S. 45; Birnbaum: Arch. f. Gyn., VII., S. 352; A. Martin: Berl. Klin. Woch., 1876, No. 28.

† Rodenstein: Amer. Jour. of Obstet, Vol. III., page 577.

‡ M. f. G., Bd. 23, S. 333.

§ Tagebl. d. Leipz. Naturf. Vers, 1872, S. 179.

wound; and Robert Barnes* proposes a very ingenious, though somewhat involved, method for doing this, so that the sutures may be entirely removed later.

These complicated procedures are unnecessary, however, as the results of ovariotomies show that silk sutures may be suffered to remain *in situ* as *ligatures perdues.*

After careful and thorough cleansing of the abdominal cavity, the parietal incision is closed with silk or metallic sutures, and a dressing of powdered iodoform with antiseptic gauze or cotton is applied. The after-treatment is the same as in ovariotomy.

The mortality of the Cæsarian operation has been variously estimated from 54 to 67 per cent.

Dr. Robert P. Harris, of Philadelphia, finds that the Cæsarian section was performed 60 times in the United States up to 1872 resulting in the saving of thirty-two women and twenty-seven children. In seventeen cases labor had been in progress for periods varying from a few hours up to twenty-four. The repetition of the operation is attended with diminished risk. Thus, of twenty-eight cases, collected by Dr. F. Churchill, in which the operation had been repeated, four died; three after the second operation, and one after the third.

In one instance, the operation was performed five times; in another, six times; and, in a third, seven times with entire safety to the offspring. What, then, was the cause of this high rate of mortality?

Of 147 fatal cases, analyzed by Dr. Chas. West, 33 perished of shock, 13 of hemorrhage, and 56 of peritonitis or metro-peritonitis; 69 cases, then, or nearly 50 per cent., died from loss of blood and the inflammation following the escape of septic fluids into the abdominal cavity, or their absorption by the uterine venous and lymphatic systems. Gross, in speaking of the hemorrhage subsequent to the operation, says that it "may be primitive or secondary, more generally the latter, and does not admit of relief."

* Lond. Obst. Trans., XII., p. 364, and Obst. Oper., 1871, p. 328.

'Twas to diminish this frightful mortality, so largely due to the failure of the uterine sutures to perform their mission in effecting union by first intention, as well as to secure for gastro-hysterotomy the brilliant results attending Listerian ovariotomies, that Prof. E. Porro*, of Pavia, suggested, in 1876, the entire ablation of uterus and its appendages after the extraction of the child, an operation which has been followed by about 50 per cent. of recoveries.

P. Müller†, of Berne, afterwards proposed to better even these results by operating outside of the abdominal wall, that the escape of fluids in the abdominal cavity might the more thoroughly be avoided, and this so-called Müller-Porro operation is the one which is now generally employed. To illustrate this latest method, and to instance the success which attends its performance by skillful hands, under the most stringent antiseptic precautions, I take the liberty of quoting from my notes of three cases, which were operated upon by Prof. Carl Braun von Fernwald in Vienna, the patients being at term:

*Case* 1. April 25, 1883. Pat. æt. ca. 28; pelvis symmetrical, but of infantile proportions.

The abdomen was carefully shaved, washed with the sublimate solution (1-1000), and covered with a rubber sheet having a central slit corresponding to the proposed incision; the edges of the opening were protected by gauze wrung out in hot carbolized water. Median incision, extending from above the umbilicus to the pubes; uterus delivered through the opening, and held in a vertical position; sponges were packed about its cervical, intra-abdominal portion, to prevent intestinal protrusion, and to receive any fluids which might escape during the operation. The pedicle was next encircled, low down, by two turns of rubber tubing, some 5 mm. in diameter, and, just above, by the chain of a large-sized écraseur; uterus rapidly incised; child removed and handed to an assistant, who tied the cord

* Porro: Della amputazione utero-ovarica, etc. Milano, 1876.

† Müller: Corresp. Bl. f. Schweizer Aerzte, 1878.

(child crying); while others tightened the elastic ligature and écraseur, both being retained in place by a long needle passed through the pedicle on their distal side. Uterus, with its appendages, was next removed with scissors about 4 cm. above the chain; bleeding was controlled; chain clamped, and handle of écraseur removed. Siver-wire sutures, each of which was armed with an oval lead plate, secured by two perforated shot clamped together upon the end of the wire, were carried through both lips of the abdominal wound, about 3 cm. from the edges, and at intervals of 5 cm. Sponges were now removed, peritoneal cavity cleansed, and the wires drawn taut and secured with similar lead plates and double shot. A continuous suture of silk was now passed, securing the wound both above and below the exit of pedicle, and this was fortified by a second and more superficial one.

The skin being protected with moist, carbolized cloths, a large Billroth clamp was applied above the needle, and the stump was seared off with Paquelin's thermo-cautery. The dressing was of thick strips of iodoform gauze, wrung out in carbolized water, over which loose, dry gauze, and antiseptic cotton was placed, the whole being topped off with the Lister sheet dressing,* to which lateral and perineal bands, long enough to reach around the trunk, had been attached. Thus the ecraseur chain and elastic ligature were left *in situ*, only to be removed at the first change of dressings, *i. e.*, after eight days.

*Case* 2. April 26, 1883. Pat. æt. 32, with a rachitic pelvis. Spray was used for several hours prior to the operation, but not pending the same. Abdominal section, etc., same as in yesterday's case. The rubber ligature about the pedicle was drawn snug, though remaining untied, an assistant holding the ends; the ecraseur chain being also passed around the pedicle, though not tightened. Now the uterus was quickly opened, the cord

---

* The Lister sheet dressing was made of gauze and Mackintosh, as suggested by its author, the whole being stitched together in the form of a pad.

compressed between two spring clamps, and the child removed. Ecraseur and ligature next tightened. In other respects the case was treated as in yesterday's operation.

This second case resulted unfortunately for the child, as, appended to the notes of an ovariotomy done two days later, I find this note: In the second case of sectio-Cæsarea the child survived only a short time, though crying when removed from the uterus, and it was found that death had resulted from the inhalation of amniotic fluid, the circulation having been retarded to too great a degree by the elastic ligature. Had such a state of affairs been suspected, said Braun, it is altogether probable that prompt efforts directed toward the clearing of the air-passages would have had a successful issue.

*Case* 3. May 1, 1883. Pat. æt. ca. 32, and suffering from marked pelvic distortion, the result of osteo-malacia.

The technique of the operation resembled that of the two previous cases, except that the child was held aloft with its face downwards immediately after delivery and before section of the cord, that the escape of any fluid present in the respiratory tract might be facilitated. The whole operation consumed forty minutes, of which four sufficed to open the abdomen, apply the ligatures and deliver the child.

In these three cases the mothers recovered promptly and without any serious drawbacks; while, of the children, one perished, as we have seen. Thus, out of a possible six, five lives were saved, and I see no reason why eighty per cent. of both mothers and children should not survive utero-ovarian amputation, or the Müller-Porro operation, when skill and the most stringent antiseptics are united to this end.

Two other alternatives, only, offer themselves when the degree of deformity calls for extreme measures, viz.: Embryotomy, or the "repeated cephalotripsy without traction," which is so ably supported by Pajot, in his brochure of 1863; and Laparo-elytrotomy, as revived by Thomas, independently of the previous labors of Ritgen and Baudelocque, and described in a paper read before the Medical Association of Yonkers, in 1870, with the title, "Gastro-elytrotomy, a Substitute for the Cæsarian Section." Of these, the first has never been successful in saving the life of the mother, even in the hands of its projector, when the conjugate axis was under two inches; and, of the second, I can only state my belief, in which I am supported by Schroeder, that it can never displace the Cæsarian operation; not because of its intrinsic difficulties, but from its limited adaptibility. With Garrigues, however, I believe that to our celebrated countryman "belongs the glory of having been the first to perform gastro-elytrotomy so as to extract a living child from a living mother in his first operation, and of having brought both mother and child to complete recovery in his second operation." *

"But," some will say in reply to my advocacy of the Müller-Porro operation, "its results are obtained only by the sacrifice of the child-bearing function, a mutilation which cannot be justified under any circumstances." And to all such I would answer, that while the removal of the internal generative organs from a woman, able to conceive but incapable of delivery *per vias naturales* by reason of permanent pelvic deformity, is not an ideal operation; it is still justifiable, nay, incumbent upon us in the present state of our knowledge, as it holds out the most hope for the mother, and almost guarantees the life of the child; while, from a moral point of view, it is vastly preferable to repeated embryotomy. The most desirable consummation, however, is such an improvement in the prognosis of the Cæsarian section that it may offer equally favorable chances, and thus permit the woes of the wretched beings who are exposed to such frightful alternatives to be mitigated in some degree by all the joys which are the reward of maternity.

---

* Garrigues on Gastro-elytrotomy. New York, 1878.

